# Editorial: Photodynamic Therapy as an Important Tool for Biological Breakthroughs—Photoactive Photosensitizers Applied from Cancer to Microbial Targets

**DOI:** 10.3390/ijms25010330

**Published:** 2023-12-26

**Authors:** Leandro M. O. Lourenço, Augusto C. Tomé, João P. C. Tomé

**Affiliations:** 1 LAQV-REQUIMTE, Department of Chemistry, University of Aveiro, 3810-193 Aveiro, Portugal; actome@ua.pt; 2 CQE, IMS, DEQ, Instituto Superior Técnico, Universidade de Lisboa, 1049-001 Lisboa, Portugal; jtome@tecnico.ulisboa.pt

## 1. Introduction

Photodynamic therapy (PDT) stands as an approved clinical treatment for both oncologic and nononcologic disorders. It boasts various advantages when compared to traditional oncological approaches like chemotherapy, radiotherapy, and surgery [1,2]. The generation of cytotoxic reactive oxygen species (ROS), especially singlet oxygen (^1^O_2_), through the interaction of harmless photoactivable molecules, known as photosensitizers (PS), with oxygen, brings about the demise of cancer cells while maintaining a favorable cosmetic outcome [3,4]. Notwithstanding the notable advancements achieved through PS-mediated PDT, this therapeutic approach encounters certain constraints. These include issues such as PS aggregation, limited solubility in physiological fluids, and specificity toward tissues or cells [5]. The development of innovative PS that may overcome these limitations is, thus, an important target.

Besides this, it is important to highlight that antimicrobial photodynamic therapy (aPDT) has been also explored as an innovative therapeutic approach because it can be used to inactivate a variety of microbial forms (e.g., bacteria [6,7,8], fungi [9,10,11], and viruses [12,13,14]) without causing significant damage to host tissues and without the development of resistance to the photosensitization process [15].

Lately, numerous research endeavors centered around novel molecules have been undertaken to enhance the selectivity and outcome of PDT using versatile PS [16]. These dyes exhibit the capacity to overcome the constraints associated with conventional PS. They not only enhance the solubility in biological environments but also bolster the PS uptake in target areas [17]. This indicates a substantial potential for advancements in the next iterations of PS agents [18], heralding the advent of a promising new generation of PS.

The subject of investigation, titled ‘Photodynamic Therapy as Important Tool for Biological Breakthroughs’, encompasses a collection of ten manuscripts that delve into the latest advancements within the field of PDT, focusing on its applications against cancer, bacteria, and viral targets. 

Markova and co-workers (contribution 1) explored perfluorocarbon nanoemulsions with fluorous chlorin-type PS for antitumor PDT in hypoxia conditions (Table 1, entry 1). The authors developed three versions of oxygen-rich chlorin molecules and used them to create nanoemulsions containing perfluorodecalin. These formulations included hydrophobic PS for absorbing specific light wavelengths and an oxygen carrier. These changes did not affect chlorin’s photosensitizing properties, including the generation of cytotoxic ROS. The emulsions entered colon carcinoma cells (HCT116) and accumulated in mitochondria. When these cells loaded with emulsions were illuminated, lipids underwent rapid peroxidation, and plasma membranes were compromised, resulting in cell death (photonecrosis). Significantly, in PDT conditions, the emulsions effectively sensitized cells cultured under prolonged hypoxia and those with acute hypoxia induced by oxygen depletion. Emulsions caused notably more photodamage in hypoxic conditions compared to situations without emulsions. With minimal dark cytotoxicity, these materials show promise as efficient and biocompatible tools for PDT-enhanced elimination of hypoxic cells.

Abrahamse and co-workers (contribution 2) emphasized the cytotoxic effects of combinative PDT effect using zinc(II) tetrasulfonic phthalocyanine (ZnPcS_4_) and cannabidiol (CBD) on a cervical cancer cell line (HeLa cells)—Table 1, entry 2. The authors emphasized the anticancer properties of CBD, an isolate from the *Cannabis sativa* L. plant. They found that CBD can inhibit tumor growth and its spread. To assess these effects, they conducted experiments using HeLa CC-cultured cells in vitro. These cells were exposed to varying doses of ZnPcS_4_ in combination with CBD. When HeLa CC cells received the lowest predetermined half-maximal inhibitory concentration dose (ICD_50_) of 0.125 µM ZnPcS_4_ along with 0.5 µM CBD and were subjected to laser irradiation, they exhibited a highly significant and advantageous form of cell death. However, further investigation into this promising treatment strategy is required.

Abrahamse and co-workers (contribution 3) also researched the synthesis, characterization, and PDT efficacy of the aluminum(III) chloride tetramercaptoacetate phthalocyanine (AlClPcTS_41_). They investigated the impact of AlClPcTS_41_ alone and when combined with PEGylated copper-gold bimetallic nanoparticles (PEG-CuAuNPs) as PS on colon cancer cells (Caco-2)—Table 1, entry 3. To create AlClPcTS_41_-PEG-CuAuNPs, they covalently linked AlClPcTS_41_ to PEG-CuAuNPs using an amide bond. Subsequently, they examined the subcellular uptake, cellular proliferation, and the antitumor PDT effects of AlClPcTS_41_, PEG-CuAuNPs, and AlClPcTS_41_-PEG-CuAuNPs in vitro using Caco-2 cells. Both the designed AlClPcTS_41_ and AlClPcTS_41_-PEG-CuAuNPs exhibited significant abilities to generate ROS, resulting in a substantial reduction in cell viability after PDT treatment.

Shigekawa and co-workers (contribution 4) evidenced a cell-level analysis to visualize the PDT process using porphylipoprotein and talaporphyrin sodium (Table 1, entry 4). Their research uncovered the contrasting mechanisms of PDT between two PS: porphylipoprotein (PLP), which has garnered recent attention for its potential in cancer treatment, and talaporphyrin sodium (NPe6). NPe6 was found to accumulate in lysosomes, whereas PLP was incorporated into phagosomes formed through PLP injection. In PDT, NPe6 generated ROS, leading to the production of actin filaments and stress fibers. However, with PLP, ROS generated by PDT remained confined within the phagosomes until the phagosomal membrane was disrupted. This delay influenced the initiation of Ras homolog family member A (RhoA) activation and RhoA*/Rho-associated coiled-coil containing kinases (ROCK) generation. Once the phagosomal membrane was disrupted, ROS were released, accelerating the production of actin filaments, stress fibers, and causing blebbing earlier than observed with NPe6. PLP also increased the elastic modulus of cells without RhoA activity in the early stage because phagosomes played a role in polymerizing actin filaments and forming pseudopodia. Given PLP’s high selectivity and uptake by cancer cells, a more substantial PDT effect can be anticipated by skillfully combining these discovered characteristics, particularly the rapid onset of a strong effect in the early stages.

Terreno and co-workers (contribution 5) have developed a prostate-specific membrane antigen (PSMA)-targeted probe designed for near-infrared fluorescence (NIRF)-guided surgery and PDT, with synthesis and preclinical validation as their primary focus. The researchers conjugated the PSMA-617 binding motif to a silicon(IV) phthalocyanine (designated by IRDye700DX-NHS), and this conjugation did not alter the photophysical characteristics of the fluorophore (Table 1, entry 5). The affinity of IRDye700DX-PSMA-617 toward prostate cancer (PCa) cells correlated with their PSMA expression levels. NIRF imaging demonstrated significant tumor uptake after injecting 1 or 5 nmol, resulting in a high tumor-to-muscle ratio. Flow cytometry and confocal images confirmed the co-localization of PSMA+ cells with IRDye700DX-PSMA uptake. Notably, ex vivo analysis of a tumor specimen revealed significant PSMA expression by tumor-associated macrophages, likely attributable to extracellular vesicles secreted by PSMA+ tumor cells. In PDT experiments, cell viability decreased in a concentration-dependent manner (from 75% at 10 nM to 12% at 500 nM), while the controls exhibited no cytotoxicity. This research has yielded a PSMA-targeted NIRF dye with dual imaging-PDT capabilities.

Malinowski and co-workers (contribution 6) embarked on a study exploring a method for producing glycosylated porphyrins through nucleophilic aromatic substitution (S_N_Ar), coupled with investigations into cellular uptake (Table 1, entry 6). Glycoporphyrins constitute a valuable group of compounds, particularly significant for applications in PDT and various other biomedical uses. Despite substantial advancements in this field, there is a growing need for innovative and diverse synthetic approaches to create carbohydrate–porphyrin hybrids. In this work, the authors introduced an efficient, gentle, and metal-free method for crafting glycoporphyrins. The versatility of the S_N_Ar procedure is demonstrated through 16 distinct examples. Additionally, preliminary biological assessments have been conducted to evaluate the cytotoxicity and cellular uptake of these molecules.

Hu and co-workers (contribution 7) have presented a study showcasing the precise eradication of malignant tumor cells through sinoporphyrin sodium-mediated PDT (Table 1, entry 7). In this research, human malignant glioblastoma U-118MG and melanoma A375 cells were chosen as targets. The product of the power density, irradiation time, and PS concentration was denoted as the total photodynamic parameter. This parameter was employed to investigate the mechanisms of PS sinoporphyrin sodium (DVDMS)-mediated PDT (DVDMS-PDT). The findings indicated a negative correlation between the survival rates of U-118MG and A375 cells and the total photodynamic parameter value in the curve. Remarkably, this correlation conformed to an exponential function. Furthermore, by manipulating the three parameters at random, the authors achieved controllable tumor cell-killing effects, thus validating the accuracy and reproducibility of their formula. The establishment and application of a functional relationship among PDT parameters play a pivotal role in predicting PDT outcomes and offering precise treatment.

Lourenço and co-workers (contribution 8) reported a study that evaluated the antifungal and sporicidal photodynamic activity of two tetra- and octa-β-substituted zinc(II) phthalocyanine dyes with dimethylaminopyridinium groups at the periphery and their quaternized derivatives (Table 1, entry 8). All phthalocyanine derivatives were prepared and assessed as PS for their effects on *Fusarium oxysporum* conidia. Antimicrobial photoinactivation experiments were performed with each PS at 0.1, 1, 10, and 20 µM under white light irradiation at an irradiance of 135 mW·cm^–2^, for 60 min (light dose of 486 J·cm^−2^). High aPDT efficiency was observed for the prepared PS (10 µM). The quaternized PS showed better aPDT performance than the non-quaternized ones, even at the low concentration of 1 µM, and a light dose of 486 J·cm^−2^. These cationic phthalocyanines are potent photodynamic PS for antifungal applications due to their ability to effectively inactivate resistant forms, like conidia, with low concentrations and reasonable energy doses.

Lourenço and co-workers (contribution 9) investigated the in vitro photoinactivation of *Fusarium oxysporum* conidia using light-activated ammonium phthalocyanines (Table 1, entry 9). This research examined the photodynamic antifungal and sporicidal capabilities of tetra- and octa-substituted phthalocyanines containing ammonium groups. The experiments involved conducting aPDT tests with varying PS concentrations while exposing them to white light. Both PS demonstrated high aPDT efficiency, reaching the detection limit. Among the two PS, the tetra-substituted variant proved to be the most effective, requiring the lowest concentration and the shortest irradiation time to completely inactivate the conidia (40 µM, 30 min, 243 J·cm^−2^). While the octa-substituted PS also achieved complete inactivation, it required a longer irradiation period and a higher concentration (60 µM, 60 min, 486 J·cm^−2^). These findings are significant because they indicate that these phthalocyanines exhibit potent antifungal photodynamic properties, demanding low concentrations and moderate energy doses to effectively combat resilient biological forms.

Lourenço and co-workers (contribution 10) documented their research on the PDT for combating T4-like bacteriophages, serving as a model for mammalian viruses in blood. This study investigated the antiviral effects of three cationic porphyrins (Table 1, entry 10) when subjected to white light exposure, and the outcomes were compared to the performance of the approved methylene blue (MB). Under isotonic conditions, none of the PS induced hemolysis when employing the T4-like phage as a surrogate for mammalian viruses. In the context of plasma, all porphyrins exhibited superior efficacy in aPDT compared to MB for inactivating the T4-like phage. Furthermore, the most efficient PS demonstrated a moderate inactivation rate for the T4-like phage in whole blood. Nevertheless, these porphyrins, much like MB, show promise as safe PS for photoinactivating viruses in blood plasma.

The Editors anticipate that the contributions gathered in this Research Topic will have a meaningful influence on the field by providing fresh insights into the research, development, and understanding of the use of remarkable dyes in the PDT approach.

## Figures and Tables

**Table 1:** Photoactive photosensitizers applied from cancer to microbial targets.

Entry	Photosensitizer Structures	Application
**1**	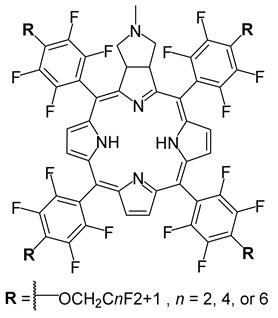	PDT in colon carcinoma cells (HCT116)
**2**	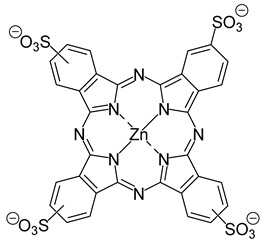	PDT in cervical cancer cell line (HeLa cells)
**3**	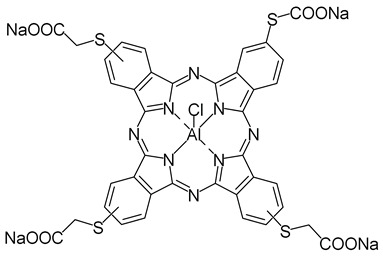	PDT in colon cancer cells (Caco-2)
**4**	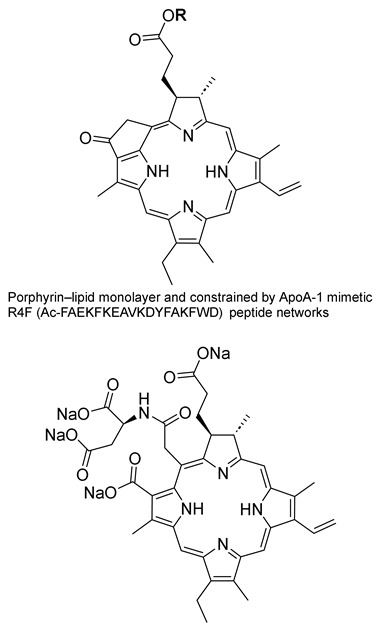	Studies in lysosomes or phagosomes
**5**	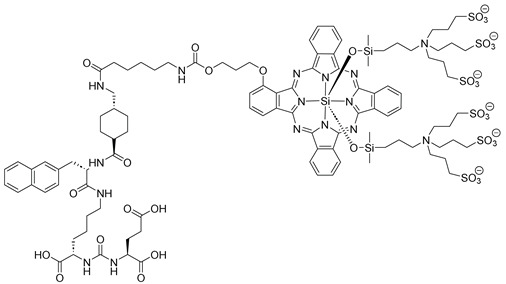	PDT in prostate cancer (PCa) cells
**6**	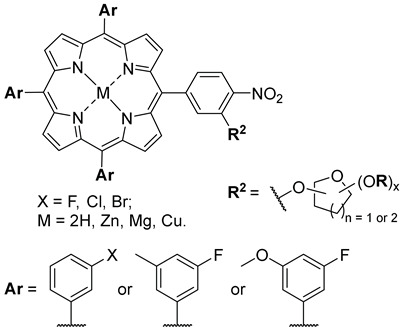	Studies in A549 and MCF-7 cellular uptake
**7**	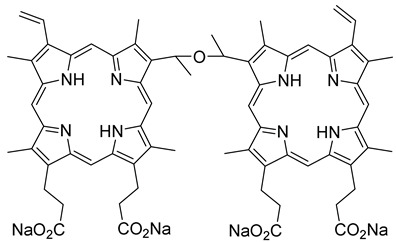	PDT in human malignant glioblastoma U-118MG and melanoma A375 cells
**8**	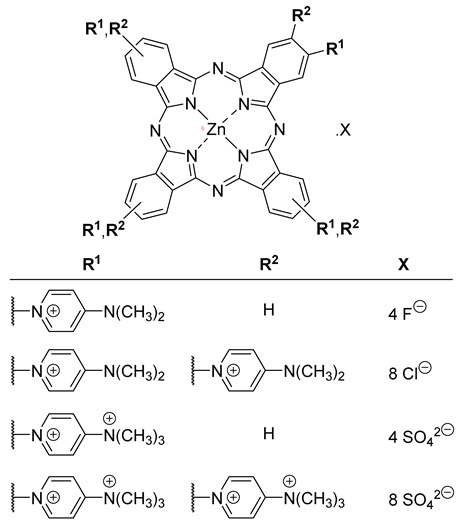	aPDT in *Fusarium* *oxysporum* conidia
**9**	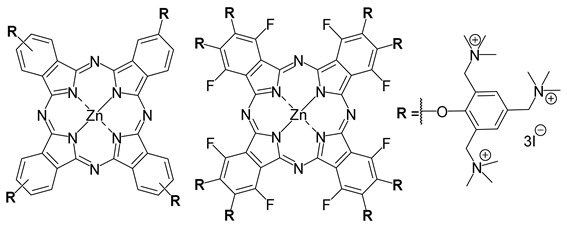	aPDT in *Fusarium* *oxysporum* conidia
**10**	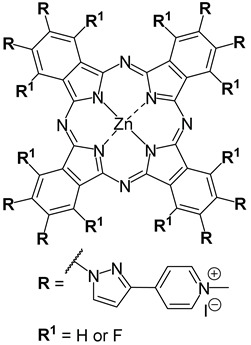	aPDT in T4-like bacteriophages
